# Influence of antibiotic treatment on the detection of *S. aureus* in whole blood following pathogen enrichment

**DOI:** 10.1186/s12866-019-1559-7

**Published:** 2019-08-06

**Authors:** Matthias Pilecky, Anita Schildberger, Ludwig Knabl, Dorothea Orth-Höller, Viktoria Weber

**Affiliations:** 1 Center for Biomedical Technology, Department for Health Sciences and Biomedicine, Danube University Krems, Dr.-Karl-Dorrek-Strasse 30, 3500 Krems, Austria; 2 Christian Doppler Laboratory for Innovative Therapy Approaches in Sepsis, Department for Health Sciences and Biomedicine, Danube University Krems, Dr.-Karl-Dorrek-Strasse 30, 3500 Krems, Austria; 3 Division of Hygiene and Medical Microbiology, Medical University of Innsbruck, Schöpfstraße 41, A-6020 Innsbruck, Austria; 4 Department for Biomedical Research, Danube University Krems, Dr.-Karl-Dorrek-Strasse 30, 3500 Krems, Austria

**Keywords:** Molecular diagnostics, Pathogen detection, Blood stream infection, DNA extraction, Selective lysis, Antibiotics

## Abstract

**Background:**

Early pathogen detection and identification are crucial for an effective and targeted antibiotic therapy in patients suffering from blood stream infection. Molecular diagnostic methods can accelerate pathogen identification as compared to blood culture, but frequently suffer from the inhibition of polymerase chain reation (PCR) by sample matrix components, such as host DNA, anticoagulants, or plasma proteins. To overcome this limitation, molecular diagnostic methods commonly rely on pathogen enrichment by selective lysis of blood cells and pelleting of intact pathogens prior to analysis.

**Results:**

Here, we investigated the impact of antibiotic treatment on the recovery of pathogen DNA using an established pathogen enrichment protocol. Based on the hypothesis that induction of bacterial cell wall disintegration following antibiotic administration leads to incomplete pelleting of pathogen DNA, *S. aureus* was grown in human whole blood with or without addition of cell wall active (vancomycin, piperacillin) or non cell wall active (ciprofloxacin, clindamycin) antibiotics at clinically relevant concentrations. Pathogen detection remained unaffected by non cell wall active antibiotics or even increased in the presence of cell wall active antibiotics, indicating improved accessibility of pathogen DNA. Likewise, mechanical lysis of *S. aureus* prior to pathogen enrichment resulted in increased recovery of pathogen DNA. Quantification of pathogen and human DNA after selective lysis of blood cells and pathogen enrichment confirmed partial depletion of human DNA, leading to a net enrichment of pathogen DNA over human DNA.

**Conclusion:**

Concurrent antibiotic administration does not reduce the recovery of pathogen DNA during pathogen enrichment by selective lysis and centrifugation.

Leads to a 10-fold human DNA depletion as compared to pathogen DNA. Moreover, we confirm that the recovery of pathogen DNA after pathogen enrichment is not negatively influenced by concurrent antibiotic administration.

**Electronic supplementary material:**

The online version of this article (10.1186/s12866-019-1559-7) contains supplementary material, which is available to authorized users.

## Background

*S. aureus* is an opportunistic pathogen with the potential to cause community-associated and nosocomial infections [1]. It is the most common gram-positive pathogen associated with sepsis, with a prevalence of up to 20% of all blood culture positive sepsis cases [2–4]. This high prevalence results from its ability to adapt to its environment by modulating the host immune response [5], to switch from a highly proliferative disseminative state into a slow growing, biofilm producing state [6], to grow intracellularly [7, 8], and to acquire antibiotic resistance [9].

Clinical guidelines for the management of sepsis [10] recommend immediate antibiotic therapy as well as pathogen identification, since early and targeted antibiotic treatment can significantly improve the survival of sepsis patients [11–14]. Blood culture is the current reference method for the identification of pathogens and for the characterization of their antibiotic susceptibility. It may, however, fail to detect slow-growing or intracellular pathogens and yield inconclusive results due to concurrent antibiotic treatment. Moreover, definitive results of culture are usually not available before 48 h [11, 13, 15]. Molecular diagnostic methods provide a time-to-result of 4–7 h, compatibility with antibiotic treatment, and do not depend on pre-selecting culture steps [13, 16, 17]. Still, the direct detection of pathogen DNA in blood samples (e.g. SeptiFast, Roche, Basel, Switzerland) is prone to interference of matrix components, such as host DNA [18, 19], heparin [20, 21], or plasma proteins [22–24]. To avoid inhibition of PCR and to increase the sensitivity of pathogen detection, virtually all current molecular diagnostic systems, including FAST ID BSI (QVella, Richmond Hill, Canada), Hybcell Pathogen Array (CubeDX, St. Valentin, Austria), T2 Bacteria (T2 Biosystems, Lexington, MA), SepsiTest (Molzym, Bremen, Germany), as well as Magicplex Sepsis Test (Seegene, Seoul, Korea), rely on pre-analytical pathogen enrichment [25]. Common to all pathogen enrichment protocols is the selective lysis of blood cells by addition of a hypotonic detergent solution. Pathogens withstand the osmotic pressure and are enriched by subsequent centrifugation prior to DNA quantification. We hypothesized that antibiotic treatment, by affecting bacterial cell wall integrity, might lead to incomplete pelleting of pathogens after selective lysis of the blood cells and centrifugation, and, consequently, result in a loss of pathogen DNA prior to analysis. Therefore, we investigated the impact of both, cell wall active and non cell wall active antibiotics on the pre-analytical enrichment of DNA of different *S. aureus* strains from human whole blood.

## Methods

### Bacteria and reagents

Antibiotics (Table 1), 4-(2-hydroxyethyl)-1-piperazineethanesulfonic acid buffer (HEPES, pH 7.0, cell culture grade) and adenine were purchased from Sigma-Aldrich (St. Louis, MO). D-Glucose was obtained from Merck (Darmstadt, Germany). *S. aureus* culture strains (ATCC 12600, 29213, and 29737) were purchased from the American Type Culture Collection (ATCC, Manassas, VA). Wild-type strains (WT32217, 32237, and 32248) were isolated from patient material at the Division of Hygiene and Medical Microbiology, Medical University of Innsbruck, Austria. Strains were cultivated on lysogeny broth (LB, Lennox formulation) agar plates (Carl Roth, Karlsruhe, Germany) at 37 °C. Overnight cultures were obtained by inoculating single colonies into LB medium. To determine the minimal inhibitory concentration for individual antibiotics used in this study, overnight cultures were diluted 1:5,000 in LB medium in 96-well polystyrene microwell plates (CELLSTAR®, Greiner Bio-One GmbH, Frickenhausen, Germany), serial dilutions of antibiotics were added, and incubation was performed for 24 h at 37 °C.

**Table 1:** Antibiotics used in this study and their mechanism of action

Antibiotic	Cell Wall Active	Mechanism of Action
Vancomycin (VAN)	+	interferes with cell wall synthesis by preventing formation and crosslinking of peptidoglycan strands [26–28]
Piperacillin (PIP)	+	interferes with cell wall synthesis by binding to enzymes required for the extracytoplasmatic stage of cell wall formation [28, 29]
Ciprofloxacin (CIP)	–	prevents replication of bacterial DNA by inhibiting DNA gyrase [30, 31]
Clindamycin (CLI)	–	inhibits bacterial protein synthesis by binding to 50S ribosomal subunits [32]

### Human whole blood

Venous human whole blood was collected from healthy adult volunteers into tubes (Vacuette, Greiner Bio-One, Kremsmuenster, Austria) containing sodium heparin or EDTA. Blood collection was approved by the Ethical Review Board of Danube University Krems, and written informed consent was obtained from all donors.

### Cultivation of *S. aureus* in human whole blood

Freshly drawn human whole blood anticoagulated with heparin was buffered with 1/50 volume of 1 M HEPES and supplemented with 2 mg/L glucose and 48 μg/L adenine per hour. Overnight cultures of each strain were diluted 1:1000 in LB medium, spiked into supplemented whole blood (typically 4 mL) at a ratio of *1:2560 (ATCC 12600), 1:205 (ATCC 29213), 1:1700 (ATCC 29737), 1:730 (WT32217), 1:1700 (WT32237), and 1:128 (WT32248),* and incubated at 37 °C with gentle shaking. These spiking ratios were chosen based on growth curves for the individual strains to obtain pathogen concentrations of approximately 5000 colony forming units (CFU) per mL after 4 h. For each strain, growth was assessed in whole blood from six donors (*n* = 3 per donor) for up to 8 h.

### Antibiotic pretreatment of *S. aureus*

To investigate whether weakening or disruption of bacterial cell walls by antibiotic pretreatment would influence subsequent pathogen enrichment and, consequently, PCR-based quantification of pathogen DNA, S. aureus was spiked into whole blood and incubated for 4 h as described above to ensure logarithmic growth. Subsequently, final concentrations of 15 μg/mL vancomycin (VAN), 20 μg/mL piperacillin (PIP), 1 μg/mL ciprofloxacin (CIP), or 2 μg/mL clindamycin (CLI), respectively (Table 1), were added, and incubation was continued for another 90 min at 37 °C with gentle agitation. Subsequent pathogen enrichment and DNA extraction were performed as described below. Spiked blood without antibiotic treatment served as control.

Strain ATCC 29213 was additionally grown in the presence of CIP and VAN for up to 72 h to investigate potential differences between blood culture based and qPCR based pathogen detection.

### Mechanical lysis of *S. aureus*

To achieve complete pathogen disintegration, fresh *S. aureus* overnight cultures (ATCC 29213) were diluted 1:5000 in LB medium, incubated for 2 h at 37 °C, mixed with an equal volume of 0.1 mm zirconium beads (Biozym, Hessisch Oldendorf, Germany) in a 0.3 mL PCR tube *(*Bio-Rad, Hercules, CA), and vortexed using a regular benchtop vortex (VortexGenie2, Carl Roth) *at* maximum speed for up to 120 min. Samples drawn after 30 s, 5 min, and 120 min were characterized by scanning electron microscopy, and bacterial lysis was confirmed by quantification of viable bacteria (CFU) as described below. Suspensions of lysed bacteria obtained at the indicated time points were spiked into freshly drawn whole blood anticoagulated with EDTA at a ratio of 1:100, and pathogen enrichment as well as isolation and quantification of pathogen DNA were performed as described below.

### Quantification of viable bacteria

To quantify viable bacteria, spiked blood samples were diluted 1:5 in 0.9% NaCl (Fresenius Kabi, Bad Homburg, Germany), and 100 μL of the dilutions were plated onto LB agar (Carl Roth, *n* = 3). Plates were incubated overnight at 37 °C, and colonies were counted manually on the next day.

### Pathogen enrichment and extraction of pathogen DNA

Pathogen DNA was isolated from spiked blood samples using a commercial pathogen enrichment system (GINA Pathogen Enrichment, CubeDX, St. Valentin, Austria) according to the instructions of the manufacturer. The enrichment protocol relies on the selective lysis of blood cells by detergent buffer, followed by centrifugation to pellet intact pathogens, lysis of pelleted pathogens by heating in alkaline buffer, and extraction of pathogen DNA using MiniSpin columns. For comparison, total DNA was extracted without previous pathogen enrichment using a total DNA extraction kit (MagMAX DNA Multi-Sample Kit, Thermo Fisher Scientific, Waltham, MA) according to the protocol of the manufacturer.

### Quantification of DNA

qPCR was performed using Staphylococcus spp. specific 16S rRNA gene primers and primers specific for the beta-actin gene as control for human DNA. All primers are specified in Additional file 1: Table S1. Samples were mixed with an equal volume of SsoAdvanced SYBR Green Master Mix (Bio-Rad, Hercules, CA) containing 0.5 μM of each primer to yield a total reaction volume of 20 μL. qPCR was performed using a Roche LightCycler 96 (Basel, Switzerland) and comprised a 2 min preheating step at 94 °C, followed by 45 cycles of denaturation at 94 °C for 5 s, annealing at 53 °C for 10 s, and extension at 72 °C for 15 s. Correct amplification was verified by melting curve analysis. cT values were calculated using the Roche LightCycler96 Software version 1.1.0.1320.

### Scanning electron microscopy

Sample preparation for scanning electron microscopy included filtration of bacterial suspensions through 0.22 μm polycarbonate isopore membranes (Merck Millipore, Darmstadt, Germany), followed by fixation with 2.5% glutaraldehyde (Carl-Roth) in 0.9% NaCl for 2 h at room temperature and dehydration in a graded alcohol series (30–100%). Samples were sputtered with gold using a Q150R (Quorum Technologies Ltd., Laughton, UK) at 30 kV in DC mode for 60 s, and images were acquired using a FlexSEM 1000 scanning electron microscope (Hitachi, Mannheim, Germany) at an accelerated voltage of 20 kV.

### Statistical analysis

Statistical analysis was performed using SigmaPlot 13.0 (Systat Software, Erkrath, Germany) and graphs were plotted using GraphPad Prism version 7.02 (La Jolla, CA). At least three replicates were performed for each experiment. Data are presented as mean ± standard deviation or as mean and 95% confidence intervals (CI). The significance of differences was calculated using paired t-test for comparison of a sample to a reference value, or, in case of multiple time points, by Friedman repeated measures analysis of variance on ranks, followed by multiple comparisons versus reference using Dunnett’s Method. *P*-values < 0.05 were considered as statistically significant.

## Results

### Growth of *S. aureus* in human whole blood and antibiotic susceptibility

**Fig. 1:**
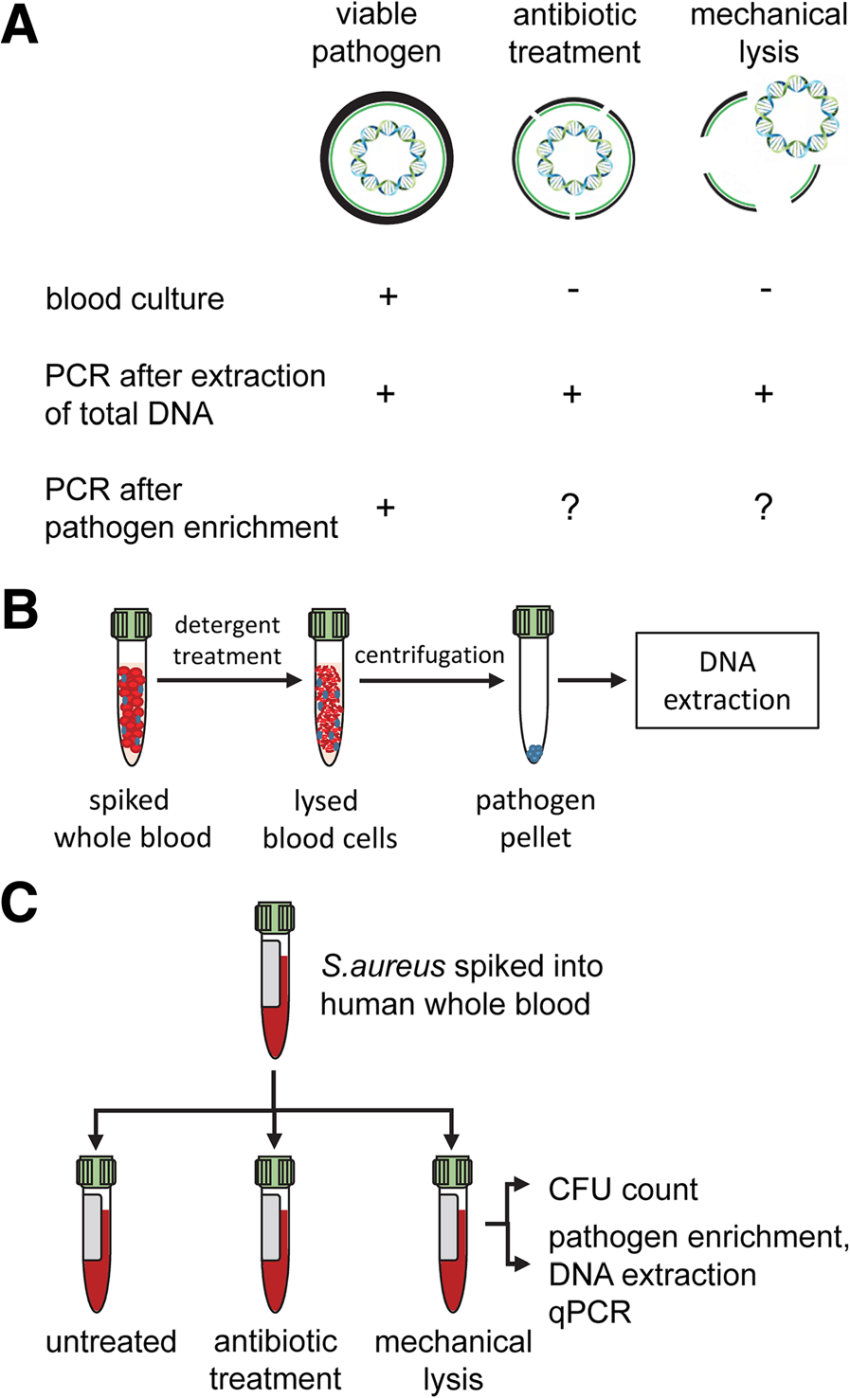
Background and outline of the study. **a** Blood-borne pathogens can be detected using blood culture (detection of viable pathogens) or by molecular diagnostic methods, such as PCR (detection of pathogen DNA). PCR is either performed after extraction of total (host and pathogen) DNA or following pathogen enrichment to deplete human DNA as well as blood-borne inhibitors of PCR. We hypothesized that antibiotic treatment might induce disintegration and, consequently, incomplete pelleting of pathogens during sample processing with established pathogen enrichment protocols, resulting in partial loss of pathogen DNA. **b** Commonly used pathogen enrichment protocols comprise the selective lysis of blood cells by detergent treatment, pelleting of intact pathogens, and extraction of pathogen DNA using spin columns. **c** To assess the influence of antibiotic treatment on PCR-based pathogen detection following pathogen enrichment, *S. aureus* was spiked into human whole blood and grown for 4 h. Thereafter, spiked samples were incubated without further treatment (control) or received antibiotic treatment as described in Materials and Methods. For comparison, *S. aureus* was mechanically lysed using zirconium beads to achieve complete disintegration as described in Materials and Methods, spiked into whole blood, and processed in parallel to untreated and antibiotic-treated samples. CFU counts (viable pathogens) were determined in all samples, and pathogen DNA was quantified after pathogen enrichment as shown in panel **b**

**Fig. 2:**
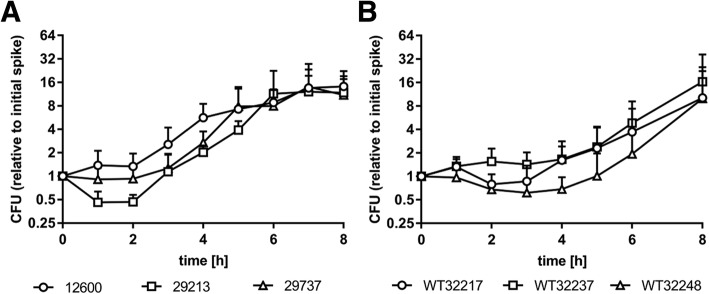
Growth of *S. aureus* in supplemented human whole blood. **a** *S. aureus* strains obtained from ATCC and **b** wild-type strains isolated from patient material reached exponential growth (log phase) after 2–4 h of incubation in whole blood supplemented with glucose and adenine as described in Materials and Methods. CFU counts are indicated relative to the initial spiking concentration of 10^3^–10^4^ CFU/mL (*n* = 6)

The experimental setup of this study is outlined in Fig. 1. All *S. aureus* strains exhibited logarithmic growth in human whole blood supplemented with glucose and adenine after a lag phase of 2–4 h (Fig. 2). All strains were susceptible to VAN, PIP, CIP and CLI. Minimal inhibitory concentrations for individual antibiotics are listed in Additional file 1: Table S2.

### Influence of antibiotic treatment on the recovery of pathogen DNA

**Fig. 3:**
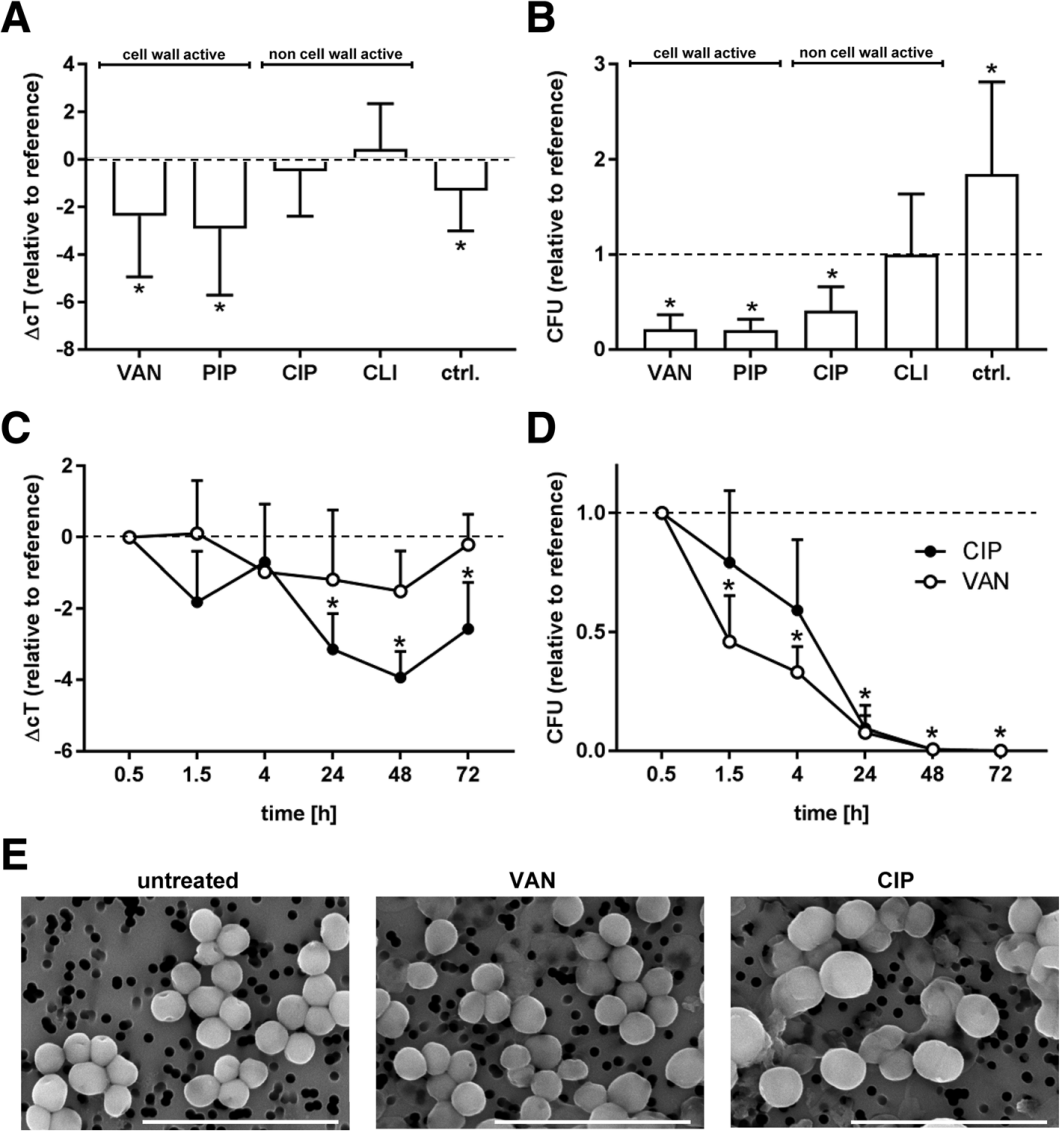
Influence of antibiotic treatment on the recovery of pathogen DNA following pathogen enrichment**. a**-**b** *S. aureus* was grown in human whole blood, followed by a 90 min incubation in the presence of antibiotics, and CFU counts as well as pathogen DNA were quantified as described in Materials and Methods. Spiked whole blood without adsorbent treatment served as control (ctrl.). ΔcT values and CFU counts are given relative to the reference (sample taken prior to the addition of antibiotics). Recovery of pathogen DNA was not influenced by CIP and CLI, while DNA recovery was increased after treatment with the cell wall active antibiotics VAN and PIP. CFU counts were significantly reduced for PIP, VAN, and CIP (bactericidal), or remained constant for CLI (bacteriostatic), confirming efficacy of antibiotic treatment. Data are presented as mean *± standard deviation* (*n* = 18; *paired t-test*). **c**-**d** Upon incubation of spiked blood samples with VAN and CIP over 72 h, the recovery of pathogen DNA remained stable (VAN) or even increased (CIP), while no viable bacteria were detectable beyond 48 h (*n* = 4; *Friedman repeated measures and Dunnett’s Method*). **e** Scanning electron micrographs of *S. aureus* incubated for 24 h in the presence of VAN and CIP failed to provide evidence for cell wall degradation or reduced culture density, as compared to the untreated control. Scale bar, 5 μm. Data are given as mean *± standard deviation*

To assess whether the presence of antibiotics would influence the recovery of *S. aureus* during pathogen enrichment, spiked whole blood was treated with either cell wall active (VAN, PIP) or non cell wall active (CIP, CLI) antibiotics (Table 1). cT values remained constant for CIP and CLI (ΔcT -0.5 ± 0.9 and 0.4 ± 0.9 as compared to the reference taken prior to the addition of antibiotics), or decreased significantly for the cell wall active antibiotics PIP and VAN (ΔcT -2.9 ± 1.4 and − 2.4 ± 1.3) (Fig. 3a), suggesting enhanced accessibility of pathogen DNA in the presence of cell wall active antibiotics. VAN, PIP, and CIP, which exhibit bactericidal activity [33–35], induced a reduction of CFU counts as compared to the reference, while CLI exerted bacteriostatic effects with stable CFU counts (Fig. 3b). No significant differences were detected between individual *S. aureus* strains (data not shown). Upon incubation with VAN and CIP for up to 72 h, pathogen DNA remained constant in the presence of VAN or even increased in the presence of CIP, providing further evidence that antibiotic treatment does not induce a loss of pathogen DNA during pathogen enrichment (Fig. 3c). CFU counts decreased continuosly over time, and no viable bacteria were detected beyond 48 h (Fig. 3d). Scanning electron microscopy did not provide evidence for cell wall degradation or reduced density of culture (Fig. 3e).

### Influence of mechanical lysis on the recovery of pathogen DNA

**Fig. 4:**
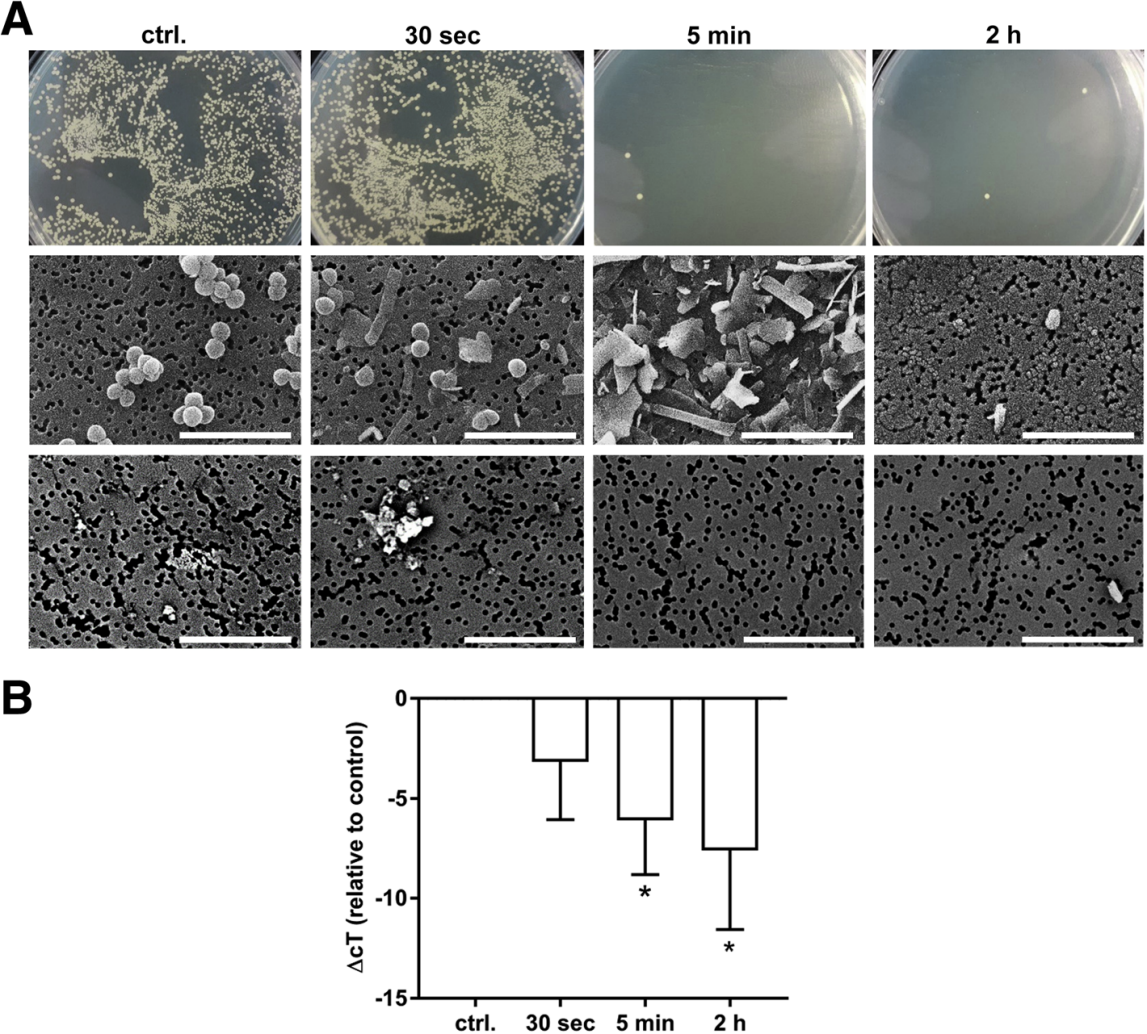
Influence of mechanical lysis on the viability and recovery of *S. aureus* DNA after pathogen enrichment. **a** *S. aureus* was mechanically lysed using zirconium beads and spiked into human whole blood as described in Materials and Methods. Culture confirmed the absence of viable bacteria after 5 min of lysis. Fragmentation of bacteria was confirmed by scanning electron microscopy. **b** Lysed *S. aureus* was spiked into whole blood, and DNA was quantified after pathogen enrichment as described in Materials and Methods. DNA recovery increased in parallel with pathogen disintegration. Data are given as mean *±* standard deviation *(n = 3;* Friedman repeated measures and Dunnett’s Method*).* Scale bar, 5 μm

Next, we performed mechanical lysis of *S.aureus* and spiked the lysates into whole blood to assess the impact of pathogen disintegration on the recovery of pathogen DNA during subsequent pathogen enrichment. Culture as well as scanning electron microscopy confirmed the efficient lysis of *S. aureus* (Fig. 4a). The recovery of pathogen DNA increased with progressing pathogen disintegration, confirming the efficient enrichment of pathogen DNA despite the absence of intact bacteria (Fig. 4b).

### Selectivity of DNA isolation: pathogen versus host DNA

Based on the enhanced recovery of pathogen DNA following pathogen disintegration by mechanical lysis, we went on to assess the ability of the pathogen enrichment protocol to selectively isolate *S. aureus* DNA, i.e. to deplete human DNA. S.aureus DNA and human DNA were quantified following DNA isolation from spiked whole blood using (i) pathogen enrichment (protocol A) or (ii) total DNA extraction without previous pathogen enrichment (protocol B). qPCR (Table 2) revealed the presence of residual human DNA in samples isolated with the pathogen enrichment protocol, however, samples obtained by total DNA extraction contained significantly higher amounts of human DNA (ΔcT 6.1 *± 0.3*). Pathogen DNA was nearly equally well enriched with both protocols (ΔcT 2.1 *± 0.4*), resulting in an approximately 16-fold enrichment of pathogen DNA over human DNA using the pathogen enrichment protocol.

**Table 2:** Selective depletion of human DNA by pathogen enrichment

		cT value	
	qPCR after	Human DNA	*S. aureus* DNA
spiked whole blood	protocol A	23.15 ± 0.38	25.98 ± 0.75
	protocol B	17.06 ± 0.08	23.85 ± 0.64

Human whole blood was spiked with *S. aureus* and processed in parallel with an estalished pathogen enrichment protocol (protocol A) and by total (host and pathogen) DNA extraction with magnetic beads (protocol B) as described in Materials and Methods. qPCR revealed the presence of residual human DNA (positive PCR for beta-actin) in samples isolated with the pathogen enrichment protocol, but samples obtained by total DNA extraction contained significantly higher amounts of human DNA. Pathogen DNA was nearly equally well enriched with both protocols, resulting in a net enrichment of pathogen DNA over host DNA using the pathogen enrichment protocol. Data are presented as mean *±* standard error of the mean (*n* = 4)

## Discussion

Early adequate antimicrobial therapy is crucial in the management of blood stream infection, where broad-spectrum antibiotics are administered upon the first suspicion of infection [10], followed by targeted antimicrobial treatment after identification of the causative pathogens by blood culture or molecular diagnostic methods [36, 37]. While there is ample evidence that antimicrobial treatment can interfere with blood culture [38, 39], the impact of antibiotic therapy on molecular diagnostic pathogen detection remains incompletely defined. The only prospective study published to date in this context revealed a significantly higher rate of positive results with SeptiFast multiplex PCR as compared to blood culture in children under concurrent antimicrobial therapy [40].

Since PCR-based pathogen detection in whole blood is, however, susceptible to inhibition by blood-borne factors and host DNA, pathogens are commonly enriched from whole blood prior to analysis. Established enrichment protocols rely on the selective lysis of blood cells in hypotonic detergent and subsequent pelleting of intact pathogens, followed by extraction of pathogen DNA and PCR. We hypothesized that antimicrobial therapy, in particular administration of cell wall active antibiotics, might result in a release of DNA from damaged bacteria and, consequently, in decreased DNA recovery during pathogen enrichment. To test this hypothesis, we chose *S. aureus* as model pathogen, since it is a leading cause of blood stream infection worldwide.

After an adaptation phase of several hours, both, *S. aureus* culture and wild-type strains reached stable growth in supplemented heparinized whole blood under our experimental conditions, while earlier studies with MRSA had observed rapid uptake of spiked *S. aureus* by neutrophils [7], with constant or decreasing CFU counts over time. The use of a methicillin-susceptible strain in our study could explain these divergent findings, since carriage of antibiotic resistance genes can reduce bacterial growth rates [41, 42]. Furthermore, we performed experiments for up to 72 h, after 4 h of adaptation of *S. aureus* to its environment. This required buffering as well as supplementation of whole blood with glucose and adenine, as commonly used for the storage of blood products [43]. Since wild type strains required a lag phase of 4 h for adaptation, we chose to incubate all strains for 4 h in supplemented whole blood prior to antibiotic administration, and we adjusted initial spiking concentrations to achieve a density of about 5000 CFU/mL after this adaptation phase. While this bacterial load was clearly higher than in septic patients, where pathogen loads of typically 1–10 CFU/mL have been reported [13], it allowed for the quantification of *S. aureus* using both, microbiological cultivation and qPCR and enabled the reliable determination of changes in qPCR signal intensity, which would not have been feasible at lower CFU counts.

To assess the influence of concurrent antibiotic treatment on pathogen enrichment, we used PIP and VAN according to clinical guidelines for the treatment of *S. aureus* infections [44, 45]. For comparison, we chose CIP, which is widely prescribed by general practitioners for the treatment of pneumonia, suspected gastrointestinal infections, or infections of the genitourinary tract [46, 47], as well as CLI, which is commonly used for the treatment of skin and soft tissue infections in outpatient care [48]. All antibiotics were applied to achieve concentrations equivalent to mean plasma levels reported in the literature [26, 27, 29–32]. We used an established manual pathogen enrichment kit, since it allows for intervention at multiple steps of the pre-analytical protocol, while protocols provided by other suppliers are fully integrated systems, which process blood samples automatically and do not allow for pathogen quantification at individual stages of the pre-analytical protocol. Still, our findings are likely to be valid for most other current pathogen enrichment protocols, as these systems differ only insignificantly regarding the composition of lysis reagents.

Regardless of the antibiotic used, *S. aureus* DNA was not lost during the pre-analytical pathogen enrichment and was still detectable after 3 days of incubation in supplemented whole blood. Scanning electron microscopy revealed that *S. aureus* stayed largely intact over the course of the experiment despite antibiotic treatment, suggesting that pathogen DNA remained encapsulated by the bacterial cell wall and was protected from blood-borne degradation factors such as DNase [49]. Notably, the administration of cell wall active antibiotics resulted in a significant increase in the qPCR signal. It is highly probable that this effect was not related to the process of pathogen enrichment as such, but was rather due to incomplete lysis of gram-positive bacteria in alkaline solution after the actual pathogen enrichment step, and we assume that cell-wall active antibiotics support the release of pathogen DNA by reducing the thickness of the peptidoglycan layer of gram-positive bacteria [50]. Even mechanical disintegration of *S. aureus* prior to pathogen enrichment resulted in enhanced detection of pathogen DNA. This further confirms previous reports on the incomplete solubilization of bacterial DNA, which was particularly reported for gram-positive bacteria [51], most likely due to its association with the cell walls [45].

While a comparison of pathogen DNA recovery from whole blood, either after selective lysis and pathogen enrichment or after extraction of total DNA, revealed that pre-analytical pathogen enrichment was not associated with enhanced detection of pathogen DNA, human DNA was depleted by 4–5 cT values, which might be relevant for the pre-analytical depletion of blood-borne PCR inhibitors. Likewise, the reduction of human DNA background may be crucial for emerging analytical approaches including next-generation sequencing [52]*.*

## Conclusion

In conclusion, our study provides evidence that concurrent antibiotic administration is not associated with decreased recovery of pathogen DNA after pathogen enrichment by selective lysis and centrifugation. On the contrary, cell wall active antibiotics seem to increase the yield of pathogen DNA, presumably by supporting pre-analytical DNA solubilization. Moreover, we confirmed a depletion of human DNA as compared to pathogen DNA by at least a factor of 10 during pathogen enrichment.

## Additional file

Additional file 1: **Table S1.** Primers for qPCR used in this study. **Table S2.** Minimal inhibitory concentrations of VAN, PIP, CIP, and CLI for the *S. aureus* strains used in this study. (PDF 292 kb)

## Data Availability

All data are presented in the manuscript and in the additional supporting material.

## References

[1] Muenks CE, Hogan PG, Wang JW, Eisenstein KA, Burnham CD, Fritz SA (2016). Diversity of Staphylococcus aureus strains colonizing various niches of the human body. J Inf Secur.

[2] Laupland KB, Lyytikainen O, Sogaard M, Kennedy KJ, Knudsen JD, Ostergaard C, Galbraith JC, Valiquette L, Jacobsson G, Collignon P (2013). The changing epidemiology of Staphylococcus aureus bloodstream infection: a multinational population-based surveillance study. Clin Microbiol Infect.

[3] Mayr FB, Yende S, Angus DC (2014). Epidemiology of severe sepsis. Virulence..

[4] Lenz R, Leal JR, Church DL, Gregson DB, Ross T, Laupland KB. The distinct category of healthcare associated bloodstream infections. BMC Infect Dis. 2012;12(85) 10.1186/1471-2334-12-85. 10.1186/1471-2334-12-85 PMC3364909 22487002

[5] Koymans KJ, Vrieling M, Gorham RD, van Strijp JAG (2017). Staphylococcal immune evasion proteins: structure, function, and host adaptation. Curr Top Microbiol Immunol.

[6] Garcia-Betancur JC, Goni-Moreno A, Horger T, Schott M, Sharan M, Eikmeier J, Wohlmuth B, Zernecke A, Ohlsen K, Kuttler C (2017). Cell differentiation defines acute and chronic infection cell types in Staphylococcus aureus. eLife..

[7] Malachowa N, Whitney AR, Kobayashi SD, Sturdevant DE, Kennedy AD, Braughton KR, Shabb DW, Diep BA, Chambers HF, Otto M (2011). Global changes in Staphylococcus aureus gene expression in human blood. PLoS One.

[8] Tuchscherr L, Heitmann V, Hussain M, Viemann D, Roth J, von Eiff C, Peters G, Becker K, Loffler B (2010). Staphylococcus aureus small-colony variants are adapted phenotypes for intracellular persistence. J Infect Dis.

[9] Monaco M, Pimentel de Araujo F, Cruciani M, Coccia EM, Pantosti A (2017). Worldwide epidemiology and antibiotic resistance of Staphylococcus aureus. Curr Top Microbiol Immunol.

[10] Rhodes A, Evans LE, Alhazzani W, Levy MM, Antonelli M, Ferrer R, Kumar A, Sevransky JE, Sprung CL, Nunnally ME (2017). Surviving Sepsis campaign: international guidelines for Management of Sepsis and Septic Shock: 2016. Crit Care Med.

[11] Fenollar F, Raoult D (2007). Molecular diagnosis of bloodstream infections caused by non-cultivable bacteria. Int J Antimicrob Agents.

[12] Garnacho-Montero J, Aldabo-Pallas T, Garnacho-Montero C, Cayuela A, Jimenez R, Barroso S, Ortiz-Leyba C (2006). Timing of adequate antibiotic therapy is a greater determinant of outcome than are TNF and IL-10 polymorphisms in patients with sepsis. Crit Care.

[13] Lamy B, Dargere S, Arendrup MC, Parienti JJ, Tattevin P (2016). How to optimize the use of blood cultures for the diagnosis of bloodstream infections? A state-of-the art. Front Microbiol.

[14] Zhang D, Micek ST, Kollef MH (2015). Time to appropriate antibiotic therapy is an independent determinant of Postinfection ICU and hospital lengths of stay in patients with Sepsis. Crit Care Med.

[15] Lamas CC, Eykyn SJ (2003). Blood culture negative endocarditis: analysis of 63 cases presenting over 25 years. Heart..

[16] Dark P, Blackwood B, Gates S, McAuley D, Perkins GD, McMullan R, Wilson C, Graham D, Timms K, Warhurst G (2015). Accuracy of LightCycler((R)) SeptiFast for the detection and identification of pathogens in the blood of patients with suspected sepsis: a systematic review and meta-analysis. Intensive Care Med.

[17] Stevenson M, Pandor A, Martyn-St James M, Rafia R, Uttley L, Stevens J, Sanderson J, Wong R, Perkins GD, McMullan R (2016). Sepsis: the LightCycler SeptiFast test MGRADE(R), SepsiTest and IRIDICA BAC BSI assay for rapidly identifying bloodstream bacteria and fungi - a systematic review and economic evaluation. Health Technol Assess.

[18] Mancini N, Carletti S, Ghidoli N, Cichero P, Burioni R, Clementi M (2010). The era of molecular and other non-culture-based methods in diagnosis of sepsis. Clin Microbiol Rev.

[19] Doring G, Unertl K, Heininger A (2008). Validation criteria for nucleic acid amplification techniques for bacterial infections. Clin Chem Lab Med.

[20] Djordjevic V, Stankovic M, Nikolic A, Antonijevic N, Rakicevic LJ, Divac A, Radojkovic M (2006). PCR amplification on whole blood samples treated with different commonly used anticoagulants. Pediatr Hematol Oncol.

[21] Garcia ME, Blanco JL, Caballero J, Gargallo-Viola D (2002). Anticoagulants interfere with PCR used to diagnose invasive aspergillosis. J Clin Microbiol.

[22] Al-Soud WA, Radstrom P (2001). Purification and characterization of PCR-inhibitory components in blood cells. J Clin Microbiol.

[23] Molina JM, Cordoba J, Ramirez P, Gobernado M (2008). Automatic detection of bacterial and fungal infections in blood. Enferm Infecc Microbiol Clin.

[24] Sidstedt M, Hedman J, Romsos EL, Waitara L, Wadso L, Steffen CR, Vallone PM, Radstrom P (2018). Inhibition mechanisms of hemoglobin, immunoglobulin G, and whole blood in digital and real-time PCR. Anal Bioanal Chem.

[25] Pilecky M, Schildberger A, Orth-Höller D, Weber V (2019). Pathogen enrichment from human whole blood for the diagnosis of bloodstream infection: prospects and limitations. Diagn Microbiol Infect Dis.

[26] Elbarbry Fawzy (2017). Vancomycin Dosing and Monitoring: Critical Evaluation of the Current Practice. European Journal of Drug Metabolism and Pharmacokinetics.

[27] Martin JH, Norris R, Barras M, Roberts J, Morris R, Doogue M, Jones GR (2010). Therapeutic monitoring of vancomycin in adult patients: a consensus review of the American Society of Health-System Pharmacists, the Infectious Diseases Society of America, and the society of infectious diseases pharmacists. Clin Biochem Rev.

[28] Sarkar P, Yarlagadda V, Ghosh C, Haldar J (2017). A review on cell wall synthesis inhibitors with an emphasis on glycopeptide antibiotics. Medchemcomm..

[29] Aardema H, Nannan Panday P, Wessels M, van Hateren K, Dieperink W, Kosterink JGW, Alffenaar JW, Zijlstra JG (2017). Target attainment with continuous dosing of piperacillin/tazobactam in critical illness: a prospective observational study. Int J Antimicrob Agents.

[30] Pierce D, Corcoran M, Martin P, Barrett K, Inglis S, Preston P, Thompson TN, Willsie SK (2014). Effect of MMX(R) mesalamine coadministration on the pharmacokinetics of amoxicillin, ciprofloxacin XR, metronidazole, and sulfamethoxazole: results from four randomized clinical trials. Drug Des Devel Ther.

[31] Szalek E, Tomczak H, Kaminska A, Grabowski T, Smuszkiewicz P, Matysiak K, Wolc A, Kaczmarek Z, Grzeskowiak E (2012). Pharmacokinetics and pharmacodynamics of ciprofloxacin in critically ill patients after the first intravenous administration of 400 mg. Adv Med Sci.

[32] Bouazza N, Pestre V, Jullien V, Curis E, Urien S, Salmon D, Treluyer JM (2012). Population pharmacokinetics of clindamycin orally and intravenously administered in patients with osteomyelitis. Br J Clin Pharmacol.

[33] Bernatova S, Samek O, Pilat Z, Sery M, Jezek J, Jakl P, Siler M, Krzyzanek V, Zemanek P, Hola V (2013). Following the mechanisms of bacteriostatic versus bactericidal action using Raman spectroscopy. Molecules..

[34] Nemeth J, Oesch G, Kuster SP (2015). Bacteriostatic versus bactericidal antibiotics for patients with serious bacterial infections: systematic review and meta-analysis. J Antimicrob Chemother.

[35] Pankey GA, Sabath LD (2004). Clinical relevance of bacteriostatic versus bactericidal mechanisms of action in the treatment of gram-positive bacterial infections. Clin Infect Dis.

[36] Carrara E, Pfeffer I, Zusman O, Leibovici L, Paul M (2018). Determinants of inappropriate empirical antibiotic treatment: systematic review and meta-analysis. Int J Antimicrob Agents.

[37] De Waele JJ, Dhaese S (2019). Antibiotic stewardship in sepsis management: toward a balanced use of antibiotics for the severely ill patient. Expert Rev Anti-Infect Ther.

[38] Grace CJ, Lieberman J, Pierce K, Littenberg B (2001). Usefulness of blood culture for hospitalized patients who are receiving antibiotic therapy. Clin Infect Dis.

[39] Scheer CS, Fuchs C, Grundling M, Vollmer M, Bast J, Bohnert JA, Zimmermann K, Hahnenkamp K, Rehberg S, Kuhn SO (2019). Impact of antibiotic administration on blood culture positivity at the beginning of sepsis: a prospective clinical cohort study. Clin Microbiol Infect.

[40] Gies F, Tschiedel E, Felderhoff-Muser U, Rath PM, Steinmann J, Dohna-Schwake C (2016). Prospective evaluation of SeptiFast multiplex PCR in children with systemic inflammatory response syndrome under antibiotic treatment. BMC Infect Dis.

[41] Knight GM, Budd EL, Lindsay JA (2013). Large mobile genetic elements carrying resistance genes that do not confer a fitness burden in healthcare-associated meticillin-resistant Staphylococcus aureus. Microbiology..

[42] Allen RC, Angst DC, Hall AR. Resistance gene carriage predicts growth of natural and clinical Escherichia coli isolates in the absence of antibiotics. Appl Environ Microbiol. 2019;85(4) 10.1128/AEM.02111-18. 10.1128/AEM.02111-18 PMC6365833 30530714

[43] D'Amici GM, Mirasole C, D'Alessandro A, Yoshida T, Dumont LJ, Zolla L (2012). Red blood cell storage in SAGM and AS3: a comparison through the membrane two-dimensional electrophoresis proteome. Blood Transfus.

[44] Liu C, Bayer A, Cosgrove SE, Daum RS, Fridkin SK, Gorwitz RJ, Kaplan SL, Karchmer AW, Levine DP, Murray BE (2011). Clinical practice guidelines by the infectious diseases society of america for the treatment of methicillin-resistant Staphylococcus aureus infections in adults and children. Clin Infect Dis.

[45] Gudiol C, Cuervo G, Shaw E, Pujol M, Carratala J (2017). Pharmacotherapeutic options for treating Staphylococcus aureus bacteremia. Expert Opin Pharmacother.

[46] Rafalsky V, Andreeva I, Rjabkova E. Quinolones for uncomplicated acute cystitis in women. Cochrane Database Syst Rev. 2006;(3):CD003597 10.1002/14651858.CD003597.pub2. 10.1002/14651858.CD003597.pub2 PMC7003573 16856014

[47] Rothberg MB, Pekow PS, Lahti M, Brody O, Skiest DJ, Lindenauer PK (2010). Comparative effectiveness of macrolides and quinolones for patients hospitalized with acute exacerbations of chronic obstructive pulmonary disease (AECOPD). J Hosp Med.

[48] Stevens DL, Bisno AL, Chambers HF, Dellinger EP, Goldstein EJ, Gorbach SL, Hirschmann JV, Kaplan SL, Montoya JG, Wade JC (2014). Practice guidelines for the diagnosis and management of skin and soft tissue infections: 2014 update by the infectious diseases society of America. Clin Infect Dis.

[49] Tamkovich SN, Cherepanova AV, Kolesnikova EV, Rykova EY, Pyshnyi DV, Vlassov VV, Laktionov PP (2006). Circulating DNA and DNase activity in human blood. Ann N Y Acad Sci.

[50] Chakraborty SP, Sahu SK, Pramanik P, Roy S (2012). In vitro antimicrobial activity of nanoconjugated vancomycin against drug resistant Staphylococcus aureus. Int J Pharm.

[51] Rantakokko-Jalava K, Jalava J (2002). Optimal DNA isolation method for detection of bacteria in clinical specimens by broad-range PCR. J Clin Microbiol.

[52] Hasan MR, Rawat A, Tang P, Jithesh PV, Thomas E, Tan R, Tilley P (2016). Depletion of human DNA in spiked clinical specimens for improvement of sensitivity of pathogen detection by next-generation sequencing. J Clin Microbiol.

