# Supplementary material for: Influence of antibiotic treatment on the detection of S. aureus in whole blood following pathogen enrichment
Source: BMC Microbiol. 2019 Aug 6;19:180. doi: 10.1186/s12866-019-1559-7 (PMC6683330; doi:10.1186/s12866-019-1559-7)
Supplement: Supplementary file 1 — Table S1. Primers for qPCR used in this study. Table S2. Minimal inhibitory concentrations of VAN, PIP, CIP, and CLI for the S. aureus strains used in this study. (PDF 292 kb) [file 12866_2019_1559_MOESM1_ESM.pdf]

Influence of antibiotic treatment on the detection of *S. aureus* DNA following pre-analytical pathogen enrichment

Supplemental Material

Matthias Pilecky[1], Anita Schildberger[1], Ludwig Knabl[3], Dorothea Orth-Höller[3] and Viktoria Weber[1,2]

[1]Center for Biomedical Technology, Department for Health Sciences and Biomedicine, Danube University Krems, Dr.-Karl-Dorrek-Strasse 30, 3500 Krems, Austria

[2]Christian Doppler Laboratory for Innovative Therapy Approaches in Sepsis, Department for Health Sciences and Biomedicine, Danube University Krems, Dr.-Karl-Dorrek-Strasse 30, 3500 Krems, Austria

[3]Division of Hygiene and Medical Microbiology, Medical University of Innsbruck, Schöpfstraße 41, A-6020 Innsbruck, Austria

Correspondence to:

Viktoria Weber, Department for Biomedical Research, Danube University Krems,

Dr.-Karl-Dorrek-Strasse 30, 3500 Krems, Austria

Tel.: +43 2732 893 2632

e-mail: viktoria.weber@donau-uni.ac.at

Table S1: Primers for qPCR used in this study.

Primer	Sequence
S. aureus forward	GTA AAA CTC TGT TAT TAG GGA AGA ACA TAT
S. aureus reverse	TAC CAG GGT ATC TAA TCC TGT TTG
human β-actin forward	CTG GAA CGG TGA AGG TGA CA
human β-actin reverse	AAG GGA CTT CCT GTA ACA ATG CA

Table S2: Minimal inhibitory concentrations of VAN, PIP, CIP, and CLI for the *S. aureus* strains used in this study.

Strain	Minimal inhibitory concentration [µg/mL]			
	VAN	PIP	CIP	CLI
12600	1.0	0.5	0.3	0.1
29213	0.5	8.0	0.3	<0.1
29737	0.3	0.1	0.1	<0.1
WT32217	0.3	0.1	0.1	<0.1
WT32237	4.0	0.3	0.5	0.1
WT32248	1.0	4.0	0.3	0.1

VAN: vancomycin, PIP: Piperacillin, CIP: Ciprofloxacin, CLI: Clindamycin
